# ^1^H NMR-MS-based heterocovariance as a drug discovery tool for fishing bioactive compounds out of a complex mixture of structural analogues

**DOI:** 10.1038/s41598-019-47434-8

**Published:** 2019-07-31

**Authors:** Ulrike Grienke, Paul A. Foster, Julia Zwirchmayr, Ammar Tahir, Judith M. Rollinger, Emmanuel Mikros

**Affiliations:** 1 Department of Pharmacognosy, Faculty of Life Sciences, University of Vienna, Althanstraße 14, 1090 Vienna, Austria; 2 Institute of Metabolism and Systems Research, University of Birmingham, Birmingham, B15 2TT United Kingdom; 3 Centre for Endocrinology, Diabetes, and Metabolism, Birmingham Health Partners, Birmingham, United Kingdom; 4 Department of Pharmacy, Division of Pharmaceutical Chemistry, School of Health Sciences, National and Kapodistrian University of Athens, Panepistimiopolis Zografou, 15771 Athens Greece

**Keywords:** Natural products, Drug discovery, Natural products

## Abstract

Chemometric methods and correlation of spectroscopic or spectrometric data with bioactivity results are known to improve dereplication in classical bio-guided isolation approaches. However, in drug discovery from natural sources the isolation of bioactive constituents from a crude extract containing close structural analogues remains a significant challenge. This study is a ^1^H NMR-MS workflow named ELINA (Eliciting Nature’s Activities) which is based on statistical heterocovariance analysis (HetCA) of ^1^H NMR spectra detecting chemical features that are positively (“hot”) or negatively (“cold”) correlated with bioactivity prior to any isolation. ELINA is exemplified in the discovery of steroid sulfatase (STS) inhibiting lanostane triterpenes (LTTs) from a complex extract of the polypore fungus *Fomitopsis pinicola*.

## Introduction

Natural products (NPs), as explained by the concept of protein fold topology^1^, cover a biologically relevant chemical space^2^ and still play a dominant role as a source for new drug leads^3,4^. However, one of the most pressing challenges remains to crack the complexity of NP extracts that will allow unravelling of the bioactive components from a mixture and make them accessible for further in-depth investigation, refinement and optimization^5^.

For many decades, the most common strategy in drug discovery from natural sources is the classical bioactivity-guided isolation. This aims to trace back the constituents responsible for activity from the crude extract by iterative chromatographic separation steps and bioactivity evaluation cycles^6^. Although prone to many disadvantages, e.g., possible degradation or loss of the active principle and the risk of overlooking minor active components, this approach is still common today^5^. Since the early 1990s, dereplication^7,8^, i.e., the early identification of known metabolites in complex mixtures to avoid their re-isolation when their bioactivity has already been described, has been integrated as a first step before starting any bioactivity-guided isolation^9^. Moreover, by focusing on the development of NMR, MS or combined approaches in combination with biochemometric tools, recent developments have led to considerable improvements of the dereplication process.

For example, the efficiency of comparing ^1^H NMR fingerprints of chromatographically obtained fractions has been demonstrated in the identification of novel drug-like natural products from an Australian marine sponge and from a termite-gut associated *Streptomyces* species^10,11^. In another study using 1D NMR data, a ^13^C NMR chemical shift database allowed dereplication via ^13^C NMR pattern recognition^12–14^. Concerning 2D NMR data, *in silico* HMBC correlation networks have been applied to assign NP structures from a mixture by using 2D molecular shift databases^15^. Additionally, approaches combining NMR with LC-HRMS data^16^, NMR with comprehensive extraction methods^17^, or molecular networking and *in silico* MS/MS fragmentation^18^, have recently emerged.

With these new techniques it is possible to dereplicate moderately complex extracts comprising diverse structural classes with different scaffolds or even very complex mixtures of the same compound class (e.g., cycloartane triterpenes, depsides)^19–21^. In latter cases, having access to or building up comprehensive databases tailor-made either for the respective structural class or NP material is a prerequisite^19^. These dereplication advances have spurred the isolation of novel scaffolds (e.g. iotrochotazine A^10^) but not necessarily bioactive ones since often the evaluation of bioactivity has not been the purpose of these studies^16^.

However recently, a paradigm shift for discovery-oriented NP chemistry has been introduced with an approach based on statistical heterocovariance analysis (HetCA)^22^. This method combines chemical and biological data of microfractions obtained after a single fractionation step performed with a bioactive crude extract. Hereby the key point is to have varying concentrations of a constituent over several fractions which correlate with a concentration dependent property, i.e., bioactivity, allowing for the early identification of bioactives in the mixture at first glance^22,23^. So far, this kind of approach is in its infancy and has only been demonstrated on artificial model mixtures^24^ or moderately complex plant extracts containing a small number of diverse low-molecular aromatic compounds^22,23^.

**Figure 1:**
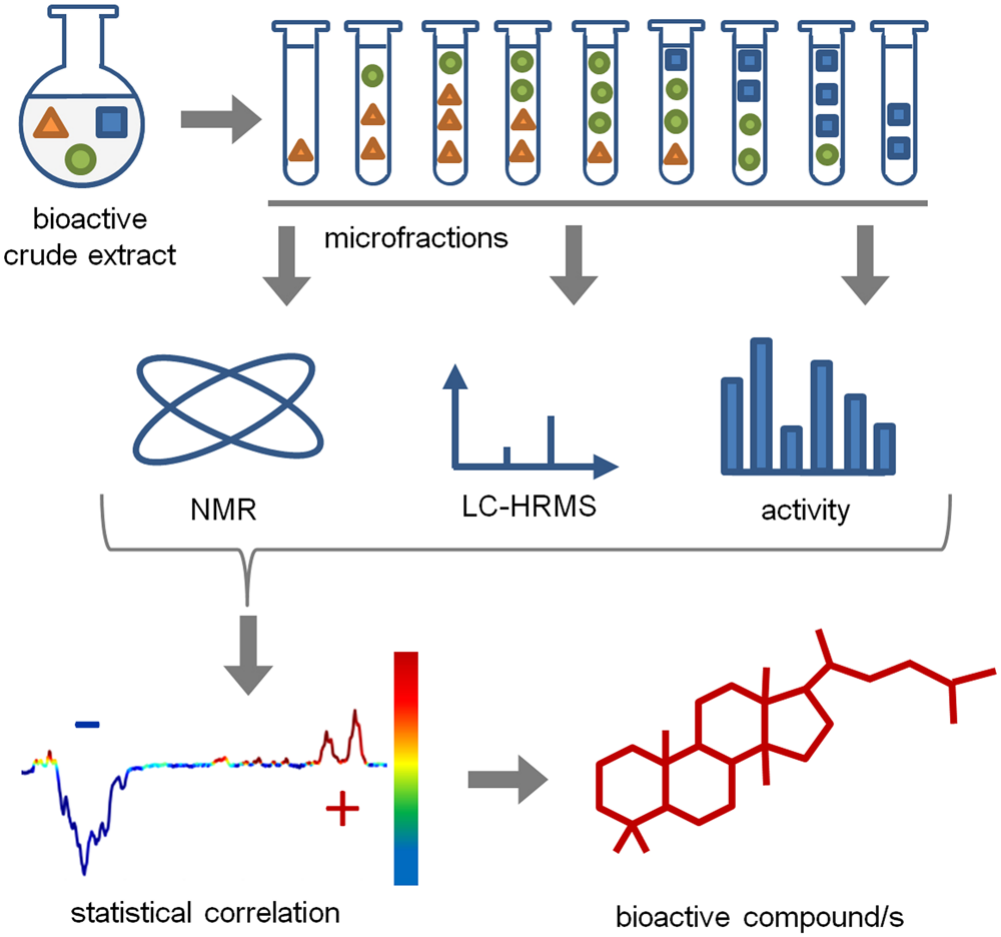
Schematic overview of the proposed “Feature Fishing” workflow. A bioactive crude extract is fractionated to create a quantitative variance of constituents over microfractions. Aliquots of these fractions are forwarded to ^1^H NMR, LC-HRESIMS, and bioactivity testing. The obtained ^1^H NMR data are statistically correlated. Based on spectroscopic regions highlighted in red and blue, relevant structural features for activity (red) can be distinguished from inactive ones (blue). Additional implementation of MS data further enables a straightforward identification and isolation of bioactive compounds.

Hit discovery from an extract containing a large number of congeners of the same structural class still resembles a Herculean task and has not been tackled with this technique so far. Being confronted with a bioactive extract of such complexity has prompted us to develop ELINA, which is based on the HetCA principle for Eliciting Nature’s Activities (Fig. 1).

The goal and purpose of ELINA is to avoid lengthy and tedious procedures involved in drug discovery from nature by:

(i) integrating high-quality chemical (NMR and MS) and biological data to provide solid criteria to prioritize targeted isolation efforts (cherry picking of compounds of particular interest) prior to any isolation.
(ii) going beyond dereplication by providing an unambiguous ^1^H NMR fingerprint of the active and inactive components in a mixture.

## Results

In an *in vitro* steroid sulfatase (STS) inhibition assay, the MeOH extract of the polypore fungus *Fomitopsis pinicola* Karst. (FP) caused 75% STS inhibition (n = 3) at a concentration of 50 *µ*g/mL. STS catalyses the conversion of sulfated precursors to free active steroids and has evolved as an attractive drug target for hormone-dependent cancers^25^. Indeed, the STS inhibitor STX64 (Irosustat) has shown clinical activity and promising results in patients with hormone-dependent breast cancer^26^. FP as well as other polypore fungi are known to mainly contain lanostane type triterpenes (LTTs) with more than 300 reported congeners endowed with many pharmacological activities^27–29^.

LTTs are usually composed of 30 carbon atoms and are characterised by a typical tetracyclic skeleton with an all *trans* configuration of the rings. The degree of oxidation and the number and position of double bonds, as well as various substituents, contribute to their structural complexity. Moreover, the analysis and detection of LTTs is often limited due to the lack of UV absorbing chromophores and low volatility resulting in difficulties in achieving sufficient ionization needed for mass spectrometry. Besides the information about the molecular weight and the presence or absence of certain distinct functional groups, MS fragmentation does not provide comprehensive structural information in the case of LTTs due to their tendency to form identical fragment patterns. Therefore, structure elucidation of LTTs strongly relies on the efficacy of 1D and 2D NMR^29^.

Aiming to unfold and simplify the mixture of LTTs present in the FP extract, time-based microfractionation via reversed phase flash chromatography was used (Fig. S1 and Table S1). In total, 125 tubes were collected and pooled to 32 microfractions according to their TLC pattern. In contrast to classical isolation efforts where the goal is to enrich single constituents in single fractions, here the final microfractions were pooled in a way to assure that constituents are deliberately spread across several fractions in varying concentrations.

Aliquots of all 32 FP microfractions were forwarded to NMR being an unbiased quantitative and therefore holistic method able to represent all constituents independent of their ability to ionize. ^1^H NMR spectra of identically prepared fraction samples were acquired under the same conditions to obtain the same signal to noise ratio. Given the rather large number of samples, ^1^H NMR was selected as a method of choice due to low acquisition times compared to ^13^C or 2D experiments. Proton resonance signals are proportional to their molar concentration allowing for a direct comparison of fractions. ^1^H NMR spectra of LTTs are characterized by almost no resonances in the downfield chemical shift area (no aromatic protons and only few double bond protons) and a rather crowded upfield area due to the greater number of aliphatic protons. This high molecular complexity of LTT spectra characterized by overlapping signals poses a great challenge for data interpretation. It clearly hinders the identification of unique spectroscopic patterns relevant within the given biological context.

In this study, a correlation of spectroscopic data reflecting the concentration differences of contained constituents with concentration-dependent activity levels was applied to simplify the spectrum leaving only the resonances of the bioactive constituents. Additional information on the investigated microfractions was collected by LC-HRESIMS analysis in the positive mode. Aliquots of the 32 FP microfractions were subjected to STS inhibition testing at a concentration of 50 *µ*g/mL. The effect of each fraction was expressed as percentage of inhibition in comparison to the positive control STX64 (Irosustat, a non-steroidal, irreversible inhibitor) which was set to 100% inhibition and the vehicle control containing 0.1% DMSO (Fig. S2). The highest activities were observed for fractions FP01_15, _16, and _17 with 67%, 64%, and 69% inhibition of the STS enzyme, respectively, whereas no inhibition was detected for the polar fractions FP01_01 to _05.

**Figure 2:**
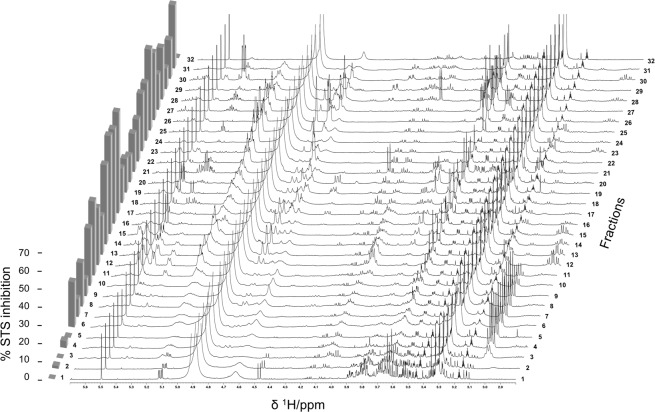
Stack plot of ^1^H NMR spectra (*δ*_H_ 2.80–5.70) of 32 FP fractions in combination with their STS inhibition profile (c = 50 *µ*g/mL) displayed as columns on the left. For clarity reasons, the methyl region is not shown.

Plotting of the carefully phased and baseline corrected ^1^H NMR spectra together with the results from the STS inhibition testing provided a first evidence of how certain resonances as well as activities fluctuate in parallel over consecutive fractions (Fig. 2). From this presentation it is obvious that ^1^H NMR chemical shifts characteristic for carbohydrate/sugar ring protons (*δ*_H_ 3.00–4.00) observed in the inactive fractions FP01_01 to _05 could rule out glycosidic molecules as STS inhibitors.

**Figure 3:**
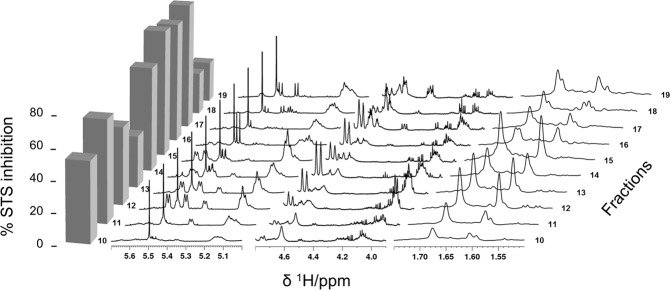
Stack plot of selected ^1^H NMR spectroscopic regions (*δ*_H_ 5.70–5.00, 4.80–3.90, and 1.75–1.50) of selected fractions FP01_10 to FP01_19 in combination with their STS inhibition profile (c = 50 *µ*g/mL) displayed as columns on the left.

Only selected regions which are well known for LTTs were useful for a simple visual inspection of the stack plotted spectra (Fig. 3), e.g., protons of double bonds at C-7 and C-11 in the triterpene skeleton (*δ*_H_ 5.70–5.00), protons of hydroxyl carrying carbons (*δ*_H_ 4.80–3.90), and methyl protons (*δ*_H_ 1.75–1.50) of the side chain attached to the C-17 of the tetracyclic scaffold. However, this requires distinct spectroscopic knowledge of the compound class under investigation. Furthermore, clear structural requirements mandatory for activity were not obvious at this point.

**Figure 4:**
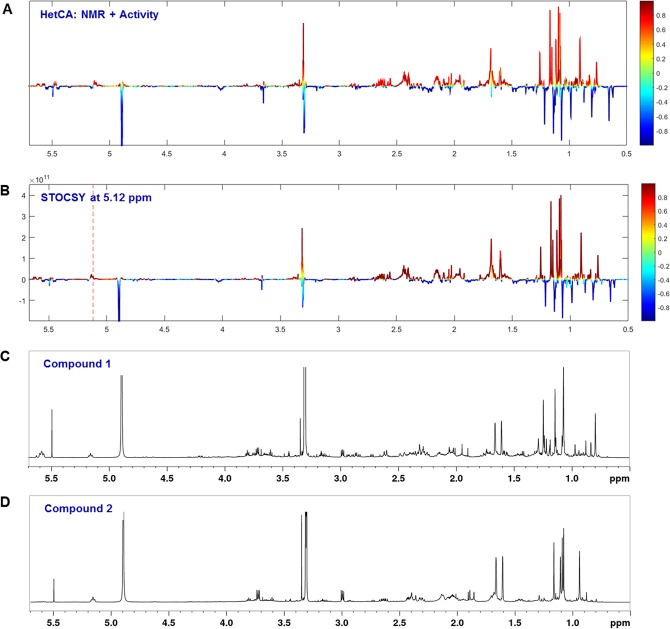
Identification of LTT features which correlate positively or negatively with STS inhibition (see Fig. S3 for zoomed area). (**A**) NMR pseudo-spectrum showing the heterocovariance (HetCA) of ^1^H NMR spectra and STS inhibition data of selected fractions FP01_13 to _15. The colour code is based on the correlation coefficient: blue = negatively correlated with STS inhibition; red = positively correlated with STS inhibition. (**B**) Statistical total correlation spectroscopy (STOCSY) plot. The signal at *δ*_H_ 5.12 was chosen to obtain the information which molecule(s) share this “hot” feature. The plot is colour coded based on the correlation coefficient: blue = signals belonging to molecule(s) that do not have the signal at *δ*_H_ 5.12; red = signals belonging to molecule(s) that have the signal at *δ*_H_ 5.12. (**C**,**D**) ^1^H NMR spectra of isolated active compounds **1** and **2**, respectively.

In order to obtain a better insight into structural features correlating to STS inhibition, the multivariate statistical tool HetCA was applied as a technique to visualize the correlation between ^1^H NMR spectra and bioactivity data resulting in so-called pseudo-spectra (HetCA plots). Over the whole series of 32 samples, this correlation was carried out for “packages” of three microfractions. In the presented example, we focused on the analysis of fractions FP01_13 to _15, since this package provided a clear variance in activity levels represented by a distinct HetCA plot (Fig. 4A). The features belonging to signals showing a positive correlation with activity (red) were named “hot” and the ones belonging to signals showing a negative correlation with activity (blue) were named “cold”.

In the crowded aliphatic region between *δ*_H_ 0.6–1.8, distinct “hot” and “cold” features typical for methyl protons were observed (Figs 4A and S3A). In the downfield chemical shift area, the already mentioned structural indicators typical for double bond protons at C-7 and C-11 (*δ*_H_ 5.57) and at C-24 (*δ*_H_ 5.12) were identified as “hot”, whereas, for instance, the prominent signal at *δ*_H_ 4.05 typical for the proton of a hydroxyl carrying C-15 was identified as “cold” (Fig. 4A).

To get more structural insights into compounds endowed with “hot” features, statistical total correlation spectroscopy (STOCSY) analysis was performed. STOCSY generates a pseudo-spectrum that reflects the correlation of the intensity variability in the ^1^H NMR signals belonging to the same specific compound(s). This analysis provides evidence of multi-colinearity and thus allows for the deconvolution and extraction of the spectroscopic features which are shared by the same molecule(s) in the complex mixture.

In the present example, the spectroscopic signals which positively correlate with the “hot” feature at *δ*_H_ 5.12 (double bond proton at C-24) were elicited. Hence, in the ^1^H NMR pseudo-spectrum, the red signals give the deconvoluted ^1^H NMR fingerprint of the active component(s) in the mixture (Figs 4B and S3B).

The STOCSY at *δ*_H_ 5.12 strongly resembles the HetCA plot (compare Fig. 4A,B) which means that the red signals of this graph point towards the molecule(s) with the main contribution to the activity within fractions FP01_13 to _15. Besides the “hot” feature at *δ*_H_ 5.12, the molecule(s) of interest include(s) the features at *δ*_H_ 5.57 (double bond protons at C-7 and C-11), and several methyl protons such as at *δ*_H_ 1.60 and at *δ*_H_ 1.65 (methyl protons at C-26 and C-27) in the region between *δ*_H_ 0.6 and *δ*_H_ 1.8. From this analysis it became evident that the active molecule(s) in fraction FP01_15 is/are typical LTT(s) scaffold(s) with a specific 2-methylpropenyl moiety (methyl protons at *δ*_H_ 1.60 and *δ*_H_ 1.65; double bond proton at *δ*_H_ 5.12) which might be localized at the end of the side chain attached to C-17.

In parallel to the NMR-bioactivity correlation, information from LC-HRESIMS data of the selected fraction series FP01_13 to _15 were analysed. An overlay of the three chromatograms (Fig. S4A) allowed for the identification of LC retention times (t_R_) of nine peaks which showed the highest peak area under the curve (AUC) in the most active fraction FP01_15 (Table S2 and Fig. S5). In particular, four peaks, those at t_R_ 25.2, 25.4, 25.9, and 26.2 min (Fig. S4B), showed a continuous increase of AUC from fraction FP01_13 to _15 reflecting a correlation with STS inhibition. These four selected peaks were analyzed for the contained *m/z* values and most plausible molecular formulas (Table S3). As a result, 22 unique molecular formulas were suggested as candidates for the four peaks.

**Figure 5:**
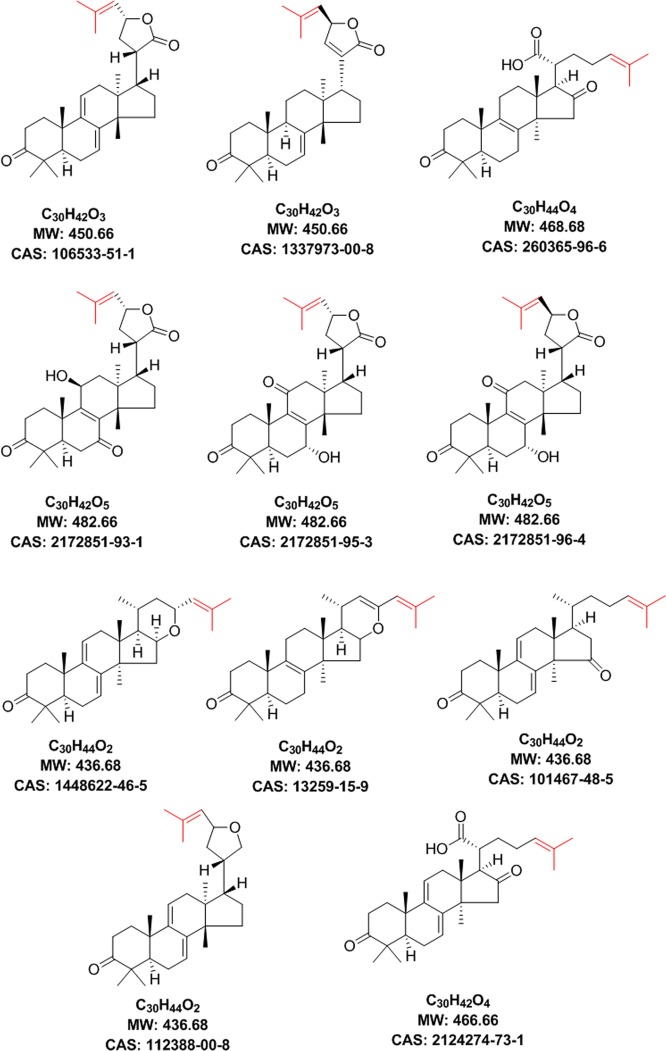
Chemical structures of eleven known candidates for Peaks I to IV after applying filters (**A–C**). The 2-methylpropenyl moiety is depicted in red colour.

To narrow down the pool of putatively active compounds, a dereplication via a literature search was performed under consideration of the “hot” and “cold” structural requirements derived from HetCA analyses. Hence, each molecular formula was searched in SciFinder^30^ (accessed 2019/01/10) and the retrieved answer set was refined by three filters: A) inclusion of compounds with the chemical substructure of an LTT backbone and the distinct 2-methylpropenyl moiety (hot feature), B) exclusion of compounds with a hydroxyl group at C-15 (cold feature), and C) exclusion of compounds with structural features not present in the HetCA pseudo-spectrum (e.g., compounds with acetyl, methylester or cyclopropyl groups). In total, the refinement using filter A resulted in 53 candidate LTTs (Figs S6–S9). By visual inspection of the structures on this list (filter B), further compounds could be excluded based on the presence of the distinct “cold” feature (i.e., hydroxyl groups at C-15). Applying filter C, by combining information from ^1^H NMR, LC-HRMS, and literature data, the number of final candidate structures was reduced to 11 known LTTs (Fig. 5 and Table S4). Taking into account that the active component(s) might as well be (a) new LTT(s) not reported in literature, underlines the possibility to apply the ELINA approach also for hitherto undisclosed constituents. In the case of a new compound, no matching structures would come up in the literature search. However, this would not hinder further isolation and structure elucidation. On the contrary, it would be even more interesting to isolate a novel bioactive compound.

**Figure 6:**
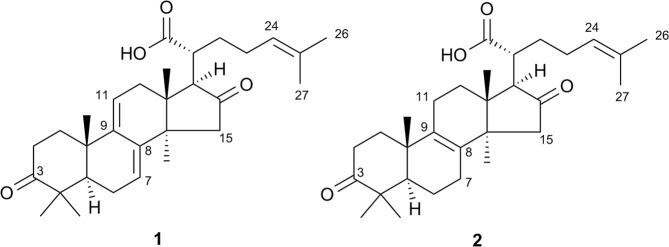
Chemical structures of active LTTs.

Based on the spectrometric and spectroscopic results, a targeted isolation of the putatively active components was performed. Therefore, we focused on the chromatographic region between t_R_ 25.2 and 26.2 min which was collected by semi-preparative reversed phase UPLC. Due to the high similarity of the contained analogues in this narrow t_R_ window and the problem of co-elution, the final isolation and purification was performed by preparative supercritical fluid chromatography as an orthogonal technique to reversed phase LC separations. In this way, two very similar LTTs (Figs 4 and 6) were selectively isolated from FP01_15 and identified by using 1D and 2D NMR experiments as well as comparison to literature data^31,32^. Piptolinic acid D (**1**) (~0.08% in the FP_M extract) has been isolated from the polypore *Piptoporus betulinus* (Bull.) P. Karst., but has never been described for FP. Pinicolic acid B (**2**) (~0.20% in the FP_M extract) has been reported previously from the fruit body of FP. Both constituents were among the eleven candidates derived from the previous literature search. These minor compounds **1** and **2** showed STS inhibiting IC_50_ values of 10.5 *µ*M and 12.4 *µ*M, respectively (Fig. S10).

When comparing their ^1^H spectroscopic data with the predicted “hot” structural indicators (Figs 4A–D and Fig. S3A–C), compound **1** has two double bond protons at *δ*_H_ 5.59 and *δ*_H_ 5.57 belonging to positions C-7 and C-11 as well as another double bond proton at *δ*_H_ 5.16 belonging to the side chain C-24; with the latter structural feature being also present in compound **2**. Both isolates contain the “hot” methyl protons at *δ*_H_ 1.61 and *δ*_H_ 1.66 located at C-25 and C-26 in the side chain of the molecules. In the methyl region between *δ*_H_ 0.6 and *δ*_H_ 1.8, the sum of the ^1^H NMR spectra of both compounds (Fig. S3C) exactly matches the STOCSY plot at *δ*_H_ 5.12 (Fig. S3B). On the other hand, “cold” features, e.g., around *δ*_H_ 4.05 characteristic for a hydroxyl carrying carbon at C-15, are not present in the isolated compounds underlining the accuracy of the performed approach.

## Discussion

Generally, the high multitude and diversity of natural product congeners is a tremendous challenge for the identification of bioactive pure constituents from a complex mixture. This refers not only to the need for sophisticated chromatography and analysis, but also to the risk of neglecting minor constituents. In this particular case of LTT congeners present in polypores like in FP, natural product researchers may profit extensively from the chemical LTT library biosynthesized by the fungal organism.

The proposed ELINA workflow makes use of the inherent orthogonality of chemical and biological data (^1^H NMR, LC-HRMS, STS inhibition). These were determined for some 30 fractions of the complex LTT mixture. Their correlations facilitated the identification of specific features which are mandatory for activity out of the pool of constituents, which in contrast to molecular databases have been made physically available by the polypore fungus.

Extending the conventional dereplication concept, the ELINA approach, linking correlation methods used in metabolomics to drug discovery, enables the fast and targeted isolation of bioactive compounds that are often overshadowed by analogues or dominant constituents. An additional strength of this procedure lies in its ability to disclose minor bioactive compounds, which would have been lost in a bioactivity-guided approach.

We believe that the ELINA approach has the potential to unravel the inherent structural complexity of a multicomponent bioactive mixtures to a target-oriented identification and isolation of novel naturally derived hit compounds in the context of drug discovery programmes.

## Methods

### General experimental procedures

NMR experiments were performed on a Bruker Avance 500 NMR spectrometer (UltraShield) (Bruker, Billerica, MA) with a 5 mm probe (TCI Prodigy CryoProbe, 5 mm, triple resonance inverse detection probe head) with z axis gradients and automatic tuning and matching accessory (Bruker BioSpin). The resonance frequency for ^1^H NMR was 500.13 MHz and for ^13^C NMR 125.75 MHz. Standard 1D and gradient-enhanced (ge) 2D experiments, like double quantum filtered (DQF) COSY, NOESY, HSQC, and HMBC, were used as supplied by the manufacturer. The calculations for HetCA and STOCSY analyses were performed using the multi-paradigm numerical computing environment MATLAB.

Liquid chromatography-High resolution mass spectrometric (LC-HRMS) analyses were performed on a Dionex Ultimate 3000 LC system hyphenated to a maXis HD ESI-Qq-TOF mass spectrometer (Bruker Daltonics, Bremen, Germany) equipped with electrospray ionization (ESI) in the positive mode: capillary voltage, 2.5 kV; nebulizer, 0.4 bar (N_2_); dry gas flow, 4 L/min (N_2_). The scanning mode was set to “auto MS/MS” operating at a mass range of 100–1500 Da with a spectra rate of 10 Hz. The sum formulas of the ions were determined using Bruker Compass DataAnalysis 4.2. Flash chromatography was performed on an Interchim puriFlash 4250 system (Montluçon, France), equipped with an evaporative light scattering detector (ELSD), a photodiode array (PDA) detector, and a fraction collector, controlled by Interchim Software. Semi-preparative supercritical fluid chromatography (SFC) was performed on a Waters Prep-15 System (Waters, Milford, MA, USA) equipped with an ELSD, a PDA detector, and a fraction collector. UPLC analysis was performed on a Waters Acquity UPLC system (H-class) equipped with a binary solvent manager, a sample manager, a column manager, a PDA detector, an ELSD, and a fraction collector.

### Fungal material

The fruit bodies of *Fomitopsis pinicola* Karst. (FP) were collected in August 2013 at 850 m in Viggartal, Ellbögen, Austria (grown on dead spruce trunk). They were dried at 40 °C for five days. The material was identified by U. Peintner (Institute of Microbiology, University of Innsbruck, Austria). Phylogenetic analyses were used to confirm the morphological identification and partial sequences of ITS rRNA were used to characterize the strain which was given the number 442^33^. A voucher specimen (no. 201300442) was deposited at the Herbarium of the Department of Pharmacognosy, University of Vienna, Austria.

### Extraction and fractionation

The dried and powdered fruit bodies of FP (~3 g) were extracted at 125 °C and 1450 psi with MeOH by using a Dionex Accelerated Solvent Extractor ASE 200 (Sunnyvale, CA). After removal of the solvent under vacuum, the MeOH extract (~950 mg, FP_M) was subjected to flash chromatography. A PuriFlash C_18_ HQ column (15 *µ*m, 35 g) was used at a flow rate of 15 mL/min applying a gradient system of MeCN/water as mobile phase (0 min 5%/95%, 30 min 45%/55%, 50 min 65%/35%, 70 min 70%/30%, 90 min 90%/20%, 115 min 98%/2%, 125 min 98%/2%) to yield 125 tubes (containing a volume of 15 mL each). These tubes were combined in such a way to ensure a minimum dry weight of 3.5 mg per fraction, sufficient for further analyses. In total, 32 microfractions (FP01_01 to FP01_32) (Fig. S1 and Table S1) were obtained. These fractions were further aliquoted to be subjected to NMR (min. dry weight: 2.25 mg) and LC-HRESIMS measurements (min. dry weight: 0.15 mg) as well as STS inhibition testing (min. dry weight: 1.00 mg).

### Targeted isolation of bioactive compounds

Fraction FP01_15 (23.6 mg) was separated by UPLC. The stationary phase was a Waters Acquity UPLC BEH C_18_ column (1.7 *µ*m, 2.1 × 100 mm) and data were analysed with Waters Empower 3. The mobile phase consisted of a 20% MeOH in MeCN/H_2_O gradient (0 min 40%/60%, 2 min 40%/60%, 45 min 99%/1%, 54 min 99%/1%). Conditions: temperature, 40 °C; flow rate, 0.3 mL/min; injection volume, 10 *µ*L; detection wavelengths, 254 nm and full range scan 190–400 nm. Three fractions (FP02_01 to FP02_03) were collected from this separation step.

Fraction FP02_01 (9 mg) was subjected to semi-preparative supercritical fluid chromatography (SFC). The stationary phase was a Torus 1-AA column (5 *µ*m, 10 mm × 250 mm) and data were analysed using MassLynx. The mobile phase consisted of a gradient system of supercritical CO_2_/MeOH:MeCN (1:1) (temperature, 50 °C; flow rate, 15 mL/min). The gradient was: 0 min 90%/10%, 7 min 85%/15%, 18 min 85%/15%, 19 min 90%/10%, 20 min 90%/10%. Six fractions (FP03_01 to FP03_06) were collected from this separation step. Fraction FP03_01 was identified as compound **1** (0.79 mg) and FP03_03 as compound **2** (1.93 mg). These two known LTTs were identified by comparing their spectroscopic and spectrometric data (1D and 2D NMR and HRESIMS) with those in the literature: piptolinic acid D (**1**)^31^ and pinicolic acid B (**2**)^32^. Copies of the original spectra for these known compounds are obtainable from the corresponding author. The purity of the isolated compounds was determined by UPLC-MS to be >98%. Fractions obtained from all chromatographic steps were analysed by TLC (mobile phase: DCM:MeOH:H_2_O, 10:1:0.25); stationary phase: Merck silica gel 60 PF_254_, detected after derivatization with vanillin/H_2_SO_4_ (5% in MeOH) under both visible light and UV_254_ and UV_366_.

### NMR analyses

The samples were measured at 298 K in fully deuterated methanol referenced to the residual non-deuterated solvent signals. Dry weighted samples (between 2.3 and 2.4 mg) of FP01_01 to FP01_32 were dissolved in methanol-d4 to reach a concentration of 3.11 mg/mL. To avoid precipitation in the NMR tube, an aliquot of 750 *µ*L of each fraction was put into an Eppendorf tube and centrifuged at 3000 rpm for 5 min. From the supernatants 645 *µ*L were transferred to NMR tubes. Standard ^1^H NMR spectra were recorded for all fractions. For the pure compounds both standard 1D and 2D experiments were performed.

### LC-HRMS data acquisition

Each fraction (c = 0.78 mg/mL in MeOH) was chromatographed over a Waters Acquity UPLC BEH C_18_ column (1.7 *µ*m, 2.1 × 100 mm) using a binary mobile phase system consisting of A) H_2_O:formic acid (100:0.02) and B) MeOH:CH_3_CN:formic acid (20:80:0.02). The gradient was from 5–95% B in 45 min followed by 15 min of column washing and re-equilibration. Conditions: temperature, 40 °C; flow rate, 0.250 mL/min; injection volume, 5 *µ*L. Samples of the isolated pure compounds were directly infused into the ESI source at a flow rate of 0.480 mL/min. Fragment ion spectra of the [M + H]^+^ or the [M + Na]^+^ ion were recorded, whereby the collision energy was manually optimized. The sum formulas of the ions were determined using Bruker Compass DataAnalysis 4.2 and isotopic pattern matching (SmartFormula algorithm): Compound **1**: C_30_H_42_O_4_ MW 466.66; Compound **2**: C_30_H_44_O_4_ MW 468.68.

### Solvents

(Ultrahigh-)gradient grade solvents from Merck (Darmstadt, Germany) and deuterated solvents from Deutero GmbH (Kastellaun, Germany) were used.

### Steroid sulfatase (STS) inhibition assay

For the FP fractions stock, solutions of 25 mg/mL in DMSO were prepared. The crude extract and the fractions were tested at a final concentration of 50 *µ*g/mL. For the isolated compounds, stock solutions of 10 mM in DMSO were prepared. Here, the final test concentration was 20 *µ*M as well as serial dilutions were tested for IC_50_ determination. STS inhibitory assays were performed as described previously^34^. Briefly, a compound’s ability to inhibit STS activity was determined using the lysate of JEG-3, a human placenta choriocarcinoma cell line known to have high STS activity. To determine STS inhibition, activity was measured in the presence of crude extract or fractions using [^3^H]E_1_S (4 × 10^5^ dpm, Perkin Elmer) adjusted to 20 *μ*M with unlabelled E_1_S substrate. After incubation of the substrate-fraction with JEG-3 lysate (125 *μ*g of protein/mL) for 1 h, the product formed was isolated from the mixture by extraction with toluene (4 mL), using [4-^14^C]E_1_ (American Radiolabeled Chemicals) to monitor procedural losses. The positive control for all experiments was 1 *µ*M of STX64 (Irosustat), a potent, selective STS inhibitor^35^.

### ^1^H NMR spectra processing and statistical correlation with bioactivity data

For all ^1^H NMR spectra of the 32 microfractions a baseline correction factor was applied using a simple polynomial curve fitting of the mathematical equation A + Bx + Cx2 + Dx3 + Ex4. Baseline correction was carried out manually using the appropriate factors^36^.

To detect structural features of the active components prior to any purification, the previously described heterocovariance (HetCA)^22,37^ analysis was applied. Briefly, ^1^H NMR spectra of relevant fraction packages were bucketed (covered range: *δ*_H_ 0.5–7.0; bucket width: 0.0005 ppm). This means that spectra were reduced in their complexity by summation of all the data points per bucket. Since each bucket width was 0.0005 ppm, this procedure gave a total of 13,000 spectroscopic buckets. The intensities of ^1^H NMR resonances of each bucket were calculated and served as variables for subsequent analyses. Covariance as a measure of the joint variability between the two variables (i) ^1^H NMR resonance intensity and (ii) percentage of STS inhibition at 50 *µ*g/mL was calculated. Additionally, the normalized version of covariance, i.e. the correlation coefficient, was calculated for colour coding.

Thus, the resulting bucket-specific covariance values were plotted as ^1^H NMR pseudo-spectrum (Fig. S3) and colour coded according to the respective correlation coefficients. This procedure allowed for the straightforward identification of ^1^H NMR resonances which are either positively (red) or negatively (blue) correlated with STS inhibition.

HetCA analysis was carried out with spectra of selected sets of fractions which showed a distinct variation in bioactivity and concentration of contained secondary metabolites, e.g. fractions FP01_13 to _15.

Additionally, as a second step, the previously established method of statistical total correlation spectroscopy (STOCSY)^22,37–39^ allowed for the detection of ^1^H NMR signals belonging to the same molecule (Fig. S3). In brief, STOCSY uses the multi-colinearity of the intensity variables over a set of spectra (e.g. the spectra of the fraction package FP01_13 to _15) to give the correlation among the intensities of the various resonances across the whole set of spectra. Here, STOCSY analysis was performed for the selected variable corresponding to *δ*_H_ 5.12 which was identified as hot feature before. The plotted ^1^H NMR pseudo-spectrum (Fig. S3) displays also covariance as a function of spectroscopic position and is colour coded according to the respective correlation coefficients (i.e. the intensities of the various resonances across the whole fraction package). Hence, STOCSY allows for the detection of multiple ^1^H NMR signals from the same molecule based on the multi-colinearity of their intensities in the selected set of ^1^H NMR spectra.

## Supplementary information

Supplementary Information

## Data Availability

The raw data generated and analysed during the current study are available from the corresponding author upon request.
