# Supplementary material for: 1H NMR-MS-based heterocovariance as a drug discovery tool for fishing bioactive compounds out of a complex mixture of structural analogues
Source: Sci Rep. 2019 Jul 31;9:11113. doi: 10.1038/s41598-019-47434-8 (PMC6668471; doi:10.1038/s41598-019-47434-8)
Supplement: Supplementary file 1 — Supplementary Information [file 41598_2019_47434_MOESM1_ESM.docx]

Supplementary Information

^1^H NMR-MS-based heterocovariance as a drug discovery tool for fishing bioactive compounds out of a complex mixture of structural analogues

Ulrike Grienke,^1*^ Paul A. Foster,^2,3^ Julia Zwirchmayr,^1^ Ammar Tahir,^1^ Judith M. Rollinger,^1^ and Emmanuel Mikros^1, 4^

^1^ Department of Pharmacognosy, Faculty of Life Sciences, University of Vienna, Althanstraße 14, 1090 Vienna, Austria

^2^ Institute of Metabolism and Systems Research, University of Birmingham, Birmingham B15 2TT, United Kingdom

^3^ Centre for Endocrinology, Diabetes, and Metabolism, Birmingham Health Partners, Birmingham, United Kingdom

^4^ Department of Pharmacy, Division of Pharmaceutical Chemistry, School of Health Sciences, National and Kapodistrian University of Athens, Panepistimiopolis Zografou, 15771, Athens, Greece

^*^ To whom the correspondence should be addressed. Phone: +43 1 4277 55262. Fax: +43 1 4277 855262. E-mail: [Ulrike.Grienke@univie.ac.at](mailto:Ulrike.Grienke@univie.ac.at)

**Index**

**Figures**

[**Figure S1.** Chromatogram of flash chromatography of FP using UV detection 245 (black) and scan (red) as well as ELSD (orange). In total, 125 tubes were collected as indicated on the top x axis. 3](#_Toc12264477)

[**Figure S2.** Percentage of STS inhibition by FP_M (crude extract) and by FP01_01 to FP01_32 (microfractions) at 50 µg/mL. STX64 (Irosustat), a potent, irreversible inhibitor of STS was used at 1 μM as positive control. 4](#_Toc12264478)

[**Figure S3.** Identification of LTT features which correlate positively or negatively with STS inhibition (upfield chemical shift area). A) NMR pseudo-spectrum showing the heterocovariance (HetCA) of ^1^H NMR spectra and STS inhibition data of selected fractions FP01_13 to _15. The colour code is based on the correlation coefficient: blue = negatively correlated with STS inhibition; red = positively correlated with STS inhibition. B) Statistical total correlation spectroscopy (STOCSY) plot. The signal at *δ*_H_ 5.12 was chosen to obtain the information which molecule(s) share this “hot” feature. The colour code is based on the correlation coefficient: blue = signals belonging to molecule(s) that do not have a signal at *δ*_H_ 5.12; red = signals belonging to molecule(s) that have a signal at *δ*_H_ 5.12. C) The overlay of the ^1^H NMR spectra of the isolated active compounds **1** and **2** matches exactly the red features of the STOCSY plot. 5](#_Toc12264479)

[**Figure S4.** LC-HRESIMS analysis. A) Overlay of mass chromatograms (positive ion mode) of fractions FP01_13 to _15. B) Four specific signals which were increasing in their intensity in the same way as the bioactivity. C) Overlay of mass chromatograms (positive ion mode) of fractions FP01_13 to _15. The peak containing the isolated compounds **1** (violet) and **2** (green) are highlighted and zoomed in. 6](#_Toc12264480)

[**Figure S5.** Graph showing peaks I to IV with an increase in peak area over the consecutive fractions FP01_13 to _15. 7](#_Toc12264481)

[**Figure S6.** Chemical structures of all candidates for peak I. Structures with a prohibition sign were excluded after applying filters A to C. 7](#_Toc12264482)

[**Figure S7.** Chemical structures of all candidates for peak II. Structures with a prohibition sign were excluded after applying filters A to C. 8](#_Toc12264483)

[**Figure S8.** Chemical structures of all candidates for peak III. Structures with a prohibition sign were excluded after applying filters A to C. 9](#_Toc12264484)

[**Figure S9.** Chemical structures of all candidates for peak IV. Structures with a prohibition sign were excluded after applying filters A to C. 10](#_Toc12264485)

[**Figure S10.** Graphs showing the concentration dependent inhibition of STS by the isolated LTTs **1**and **2** (mean ± S.D., n = 3). 10](#_Toc12264486)

**Tables**

[**Table S1.** Overview on combined FP microfractions. 11](#_Toc12093545)

[**Table S2.** Overview of retention times and peak areas that strongly correlate with activity. 12](#_Toc12093546)

[**Table S3.** Overview of retention times, *m/z* values and potential molecular formula candidates that strongly correlate with activity. 12](#_Toc12093547)

[**Table S4.** Results from the literature search in SciFinder giving the number of potential candidates for STS inhibiting LTTs after applying filters A to C. 12](#_Toc12093548)

Figures


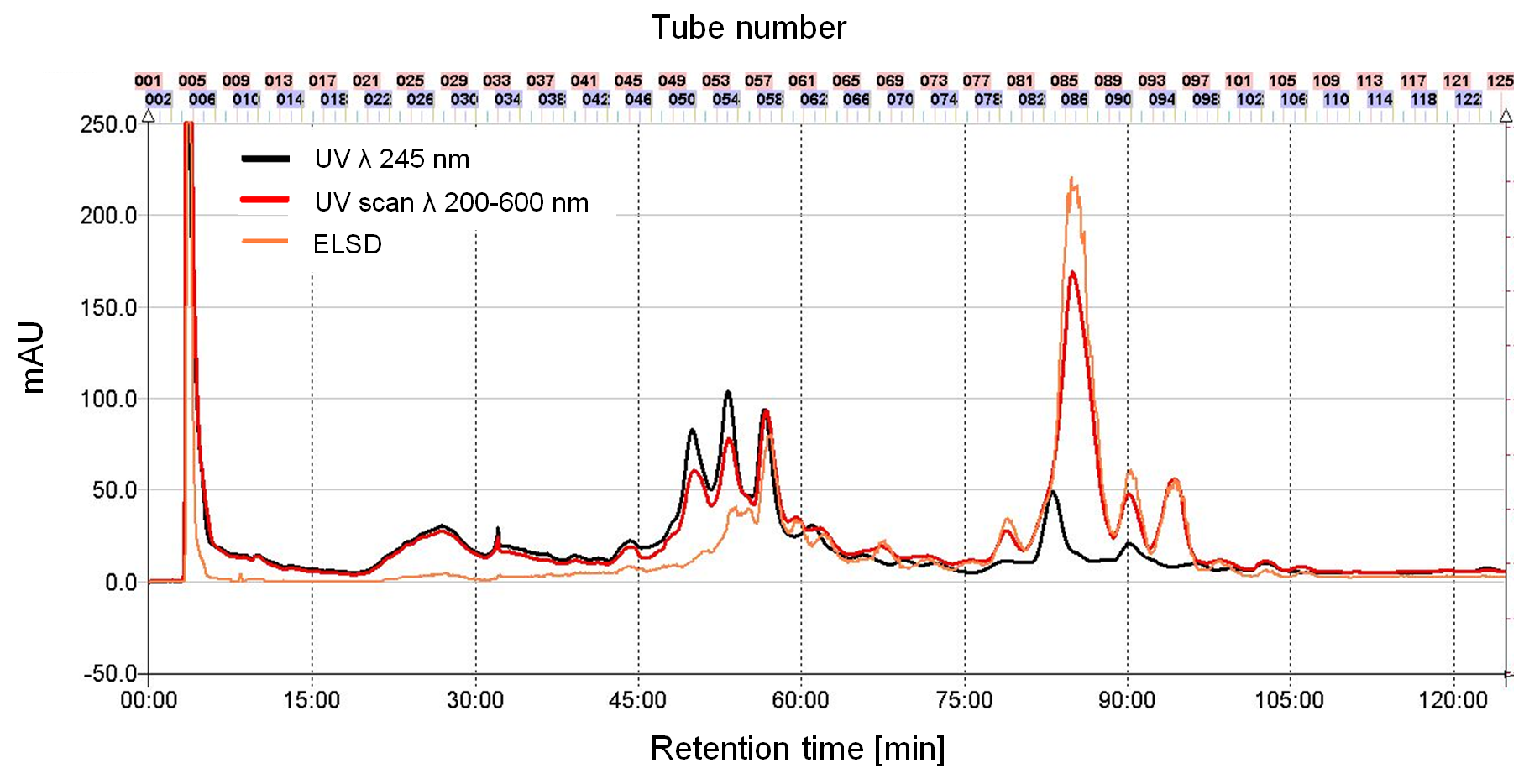


**Figure S1.** Chromatogram of flash chromatography of FP using UV detection 245 (black) and scan (red) as well as ELSD (orange). In total, 125 tubes were collected as indicated on the top x axis.

**Figure S2.** Percentage of STS inhibition by FP_M (crude extract) and by FP01_01 to FP01_32 (microfractions) at 50 µg/mL. STX64 (Irosustat), a potent, irreversible inhibitor of STS was used at 1 μM as positive control.


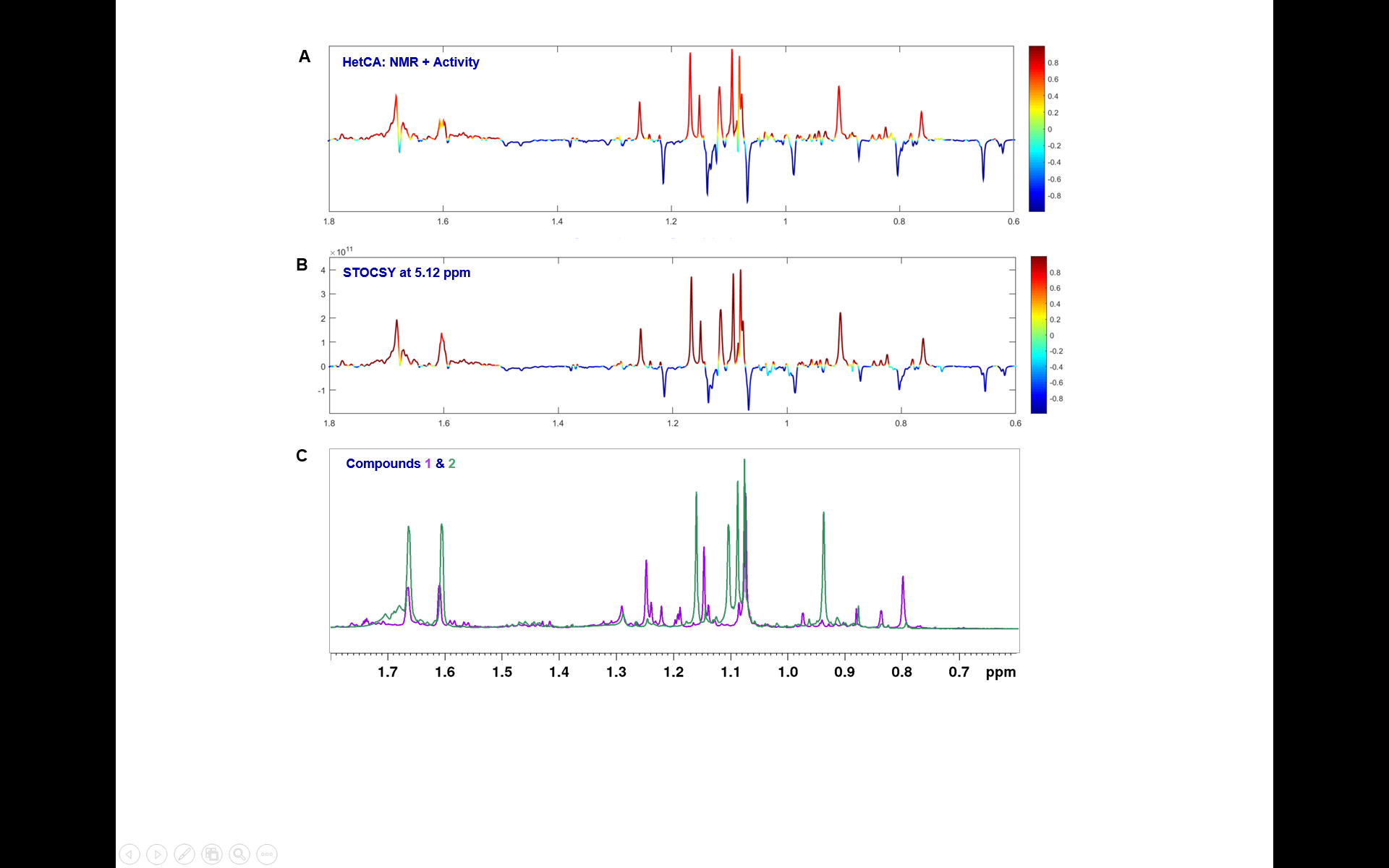


**Figure S3.** Identification of LTT features which correlate positively or negatively with STS inhibition (upfield chemical shift area). A) NMR pseudo-spectrum showing the heterocovariance (HetCA) of ^1^H NMR spectra and STS inhibition data of selected fractions FP01_13 to _15. The colour code is based on the correlation coefficient: blue = negatively correlated with STS inhibition; red = positively correlated with STS inhibition. B) Statistical total correlation spectroscopy (STOCSY) plot. The signal at *δ*_H_ 5.12 was chosen to obtain the information which molecule(s) share this “hot” feature. The colour code is based on the correlation coefficient: blue = signals belonging to molecule(s) that do not have a signal at *δ*_H_ 5.12; red = signals belonging to molecule(s) that have a signal at *δ*_H_ 5.12. C) The overlay of the ^1^H NMR spectra of the isolated active compounds **1** and **2** matches exactly the red features of the STOCSY plot.

A


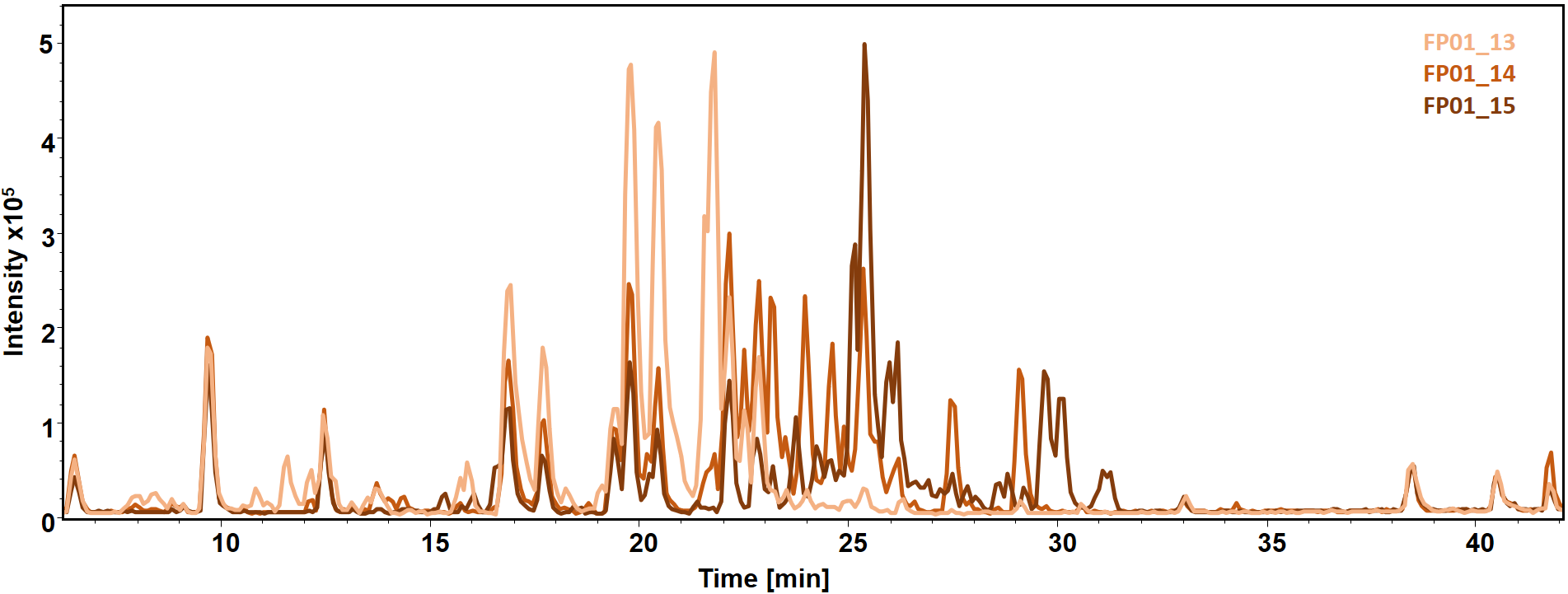


B


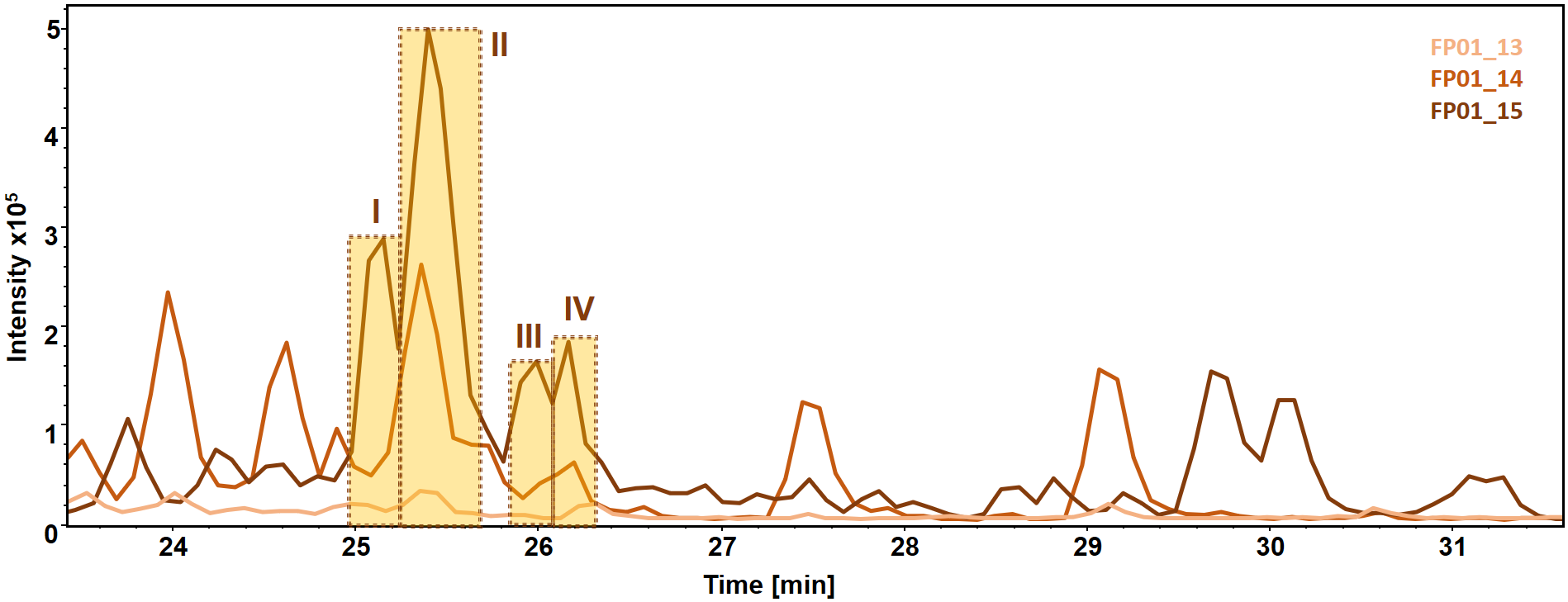


C


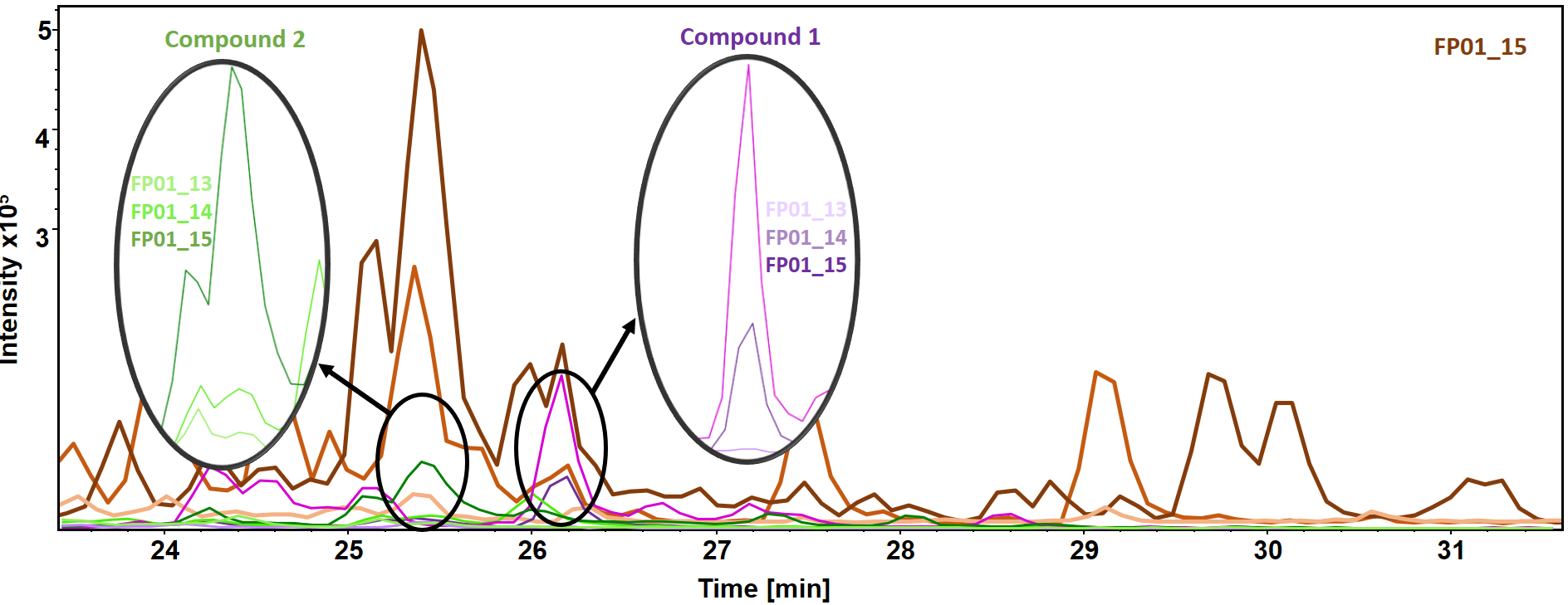


**Figure S4.** LC-HRESIMS analysis. A) Overlay of mass chromatograms (positive ion mode) of fractions FP01_13 to _15. B) Four specific signals which were increasing in their intensity in the same way as the bioactivity. C) Overlay of mass chromatograms (positive ion mode) of fractions FP01_13 to _15. The peak containing the isolated compounds **1** (violet) and **2** (green) are highlighted and zoomed in.


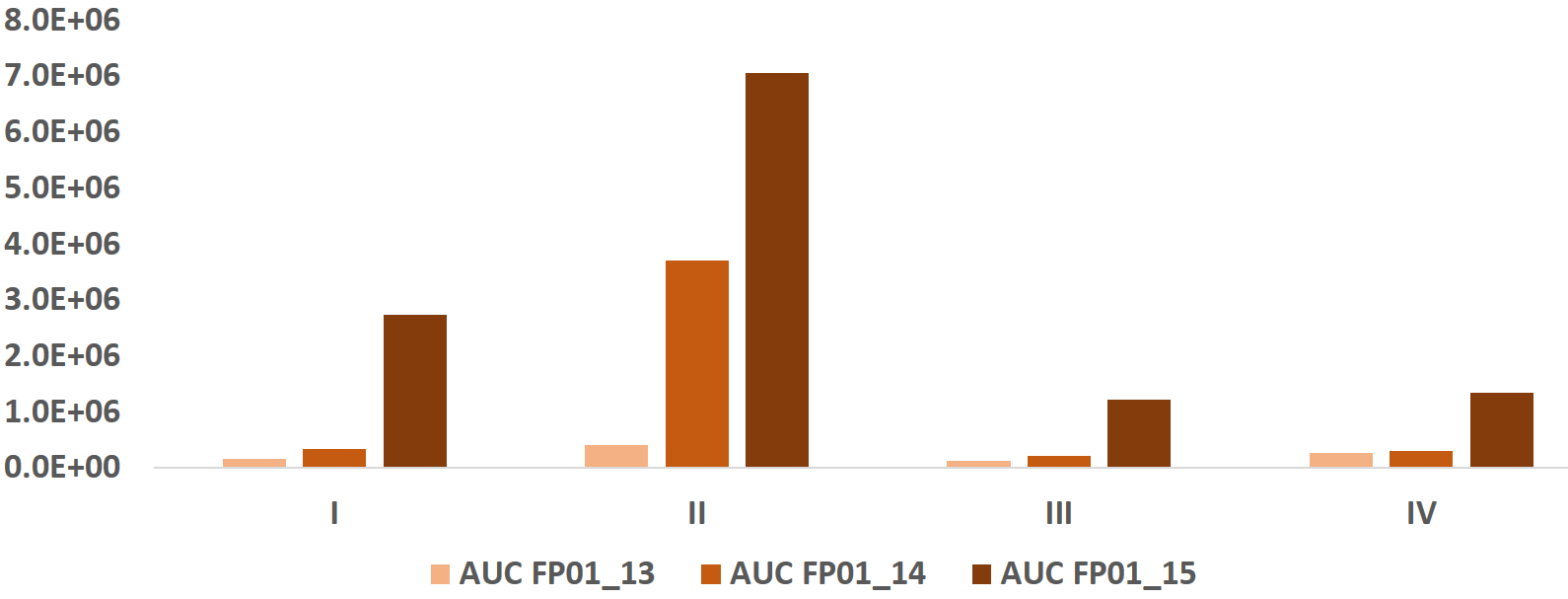


**Figure S5.** Graph showing peaks I to IV with an increase in peak area over the consecutive fractions FP01_13 to _15.


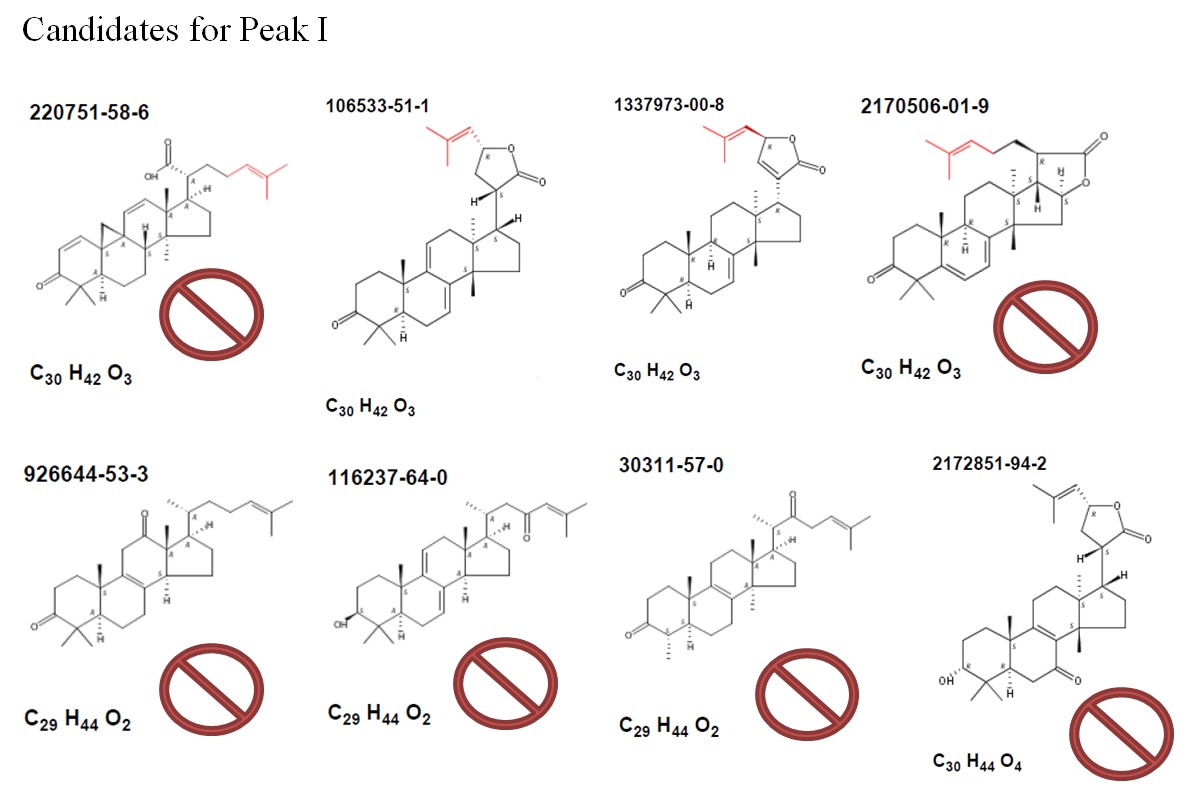

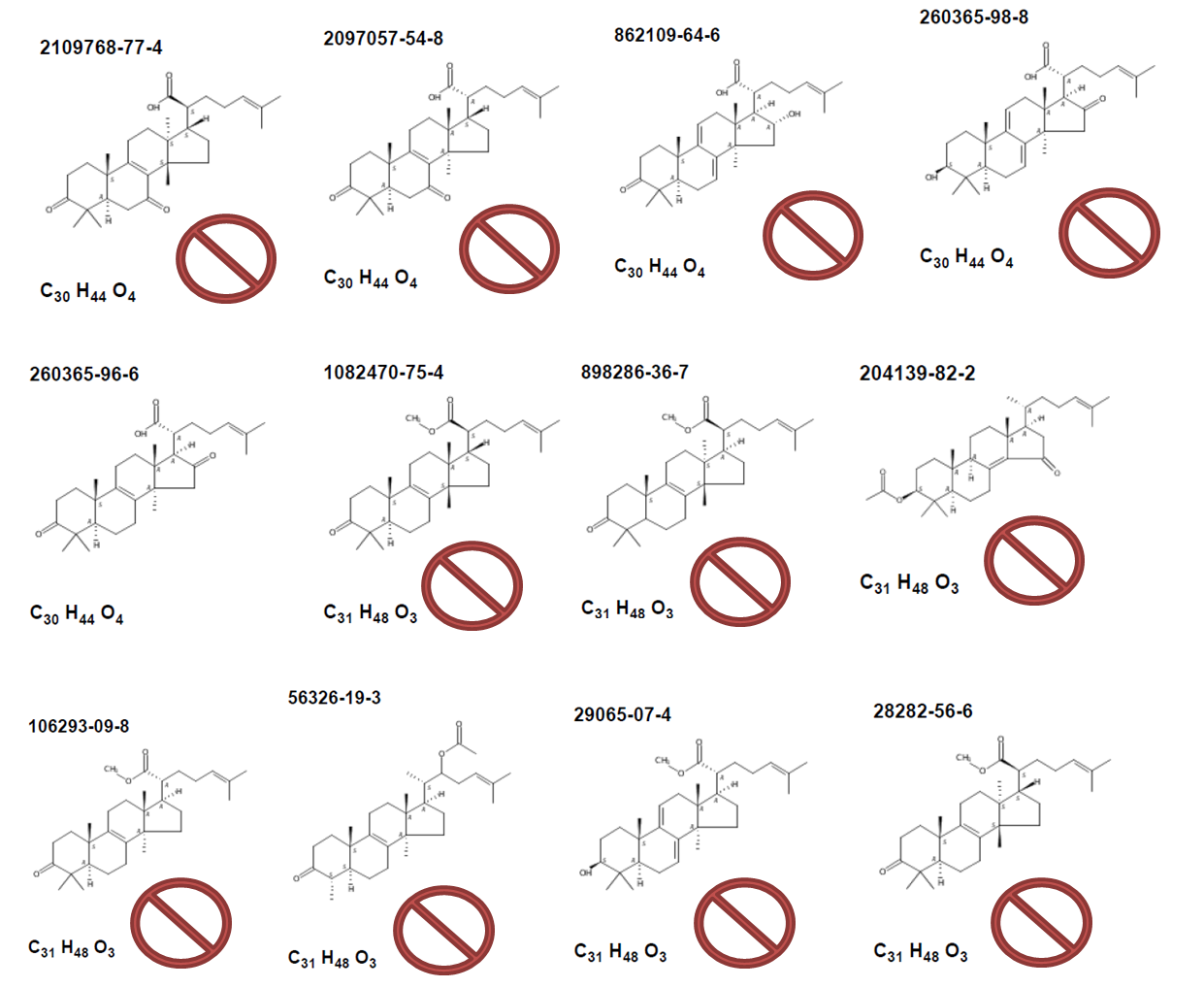


**Figure S6.** Chemical structures of all candidates for peak I. Structures with a prohibition sign were excluded after applying filters A to C.


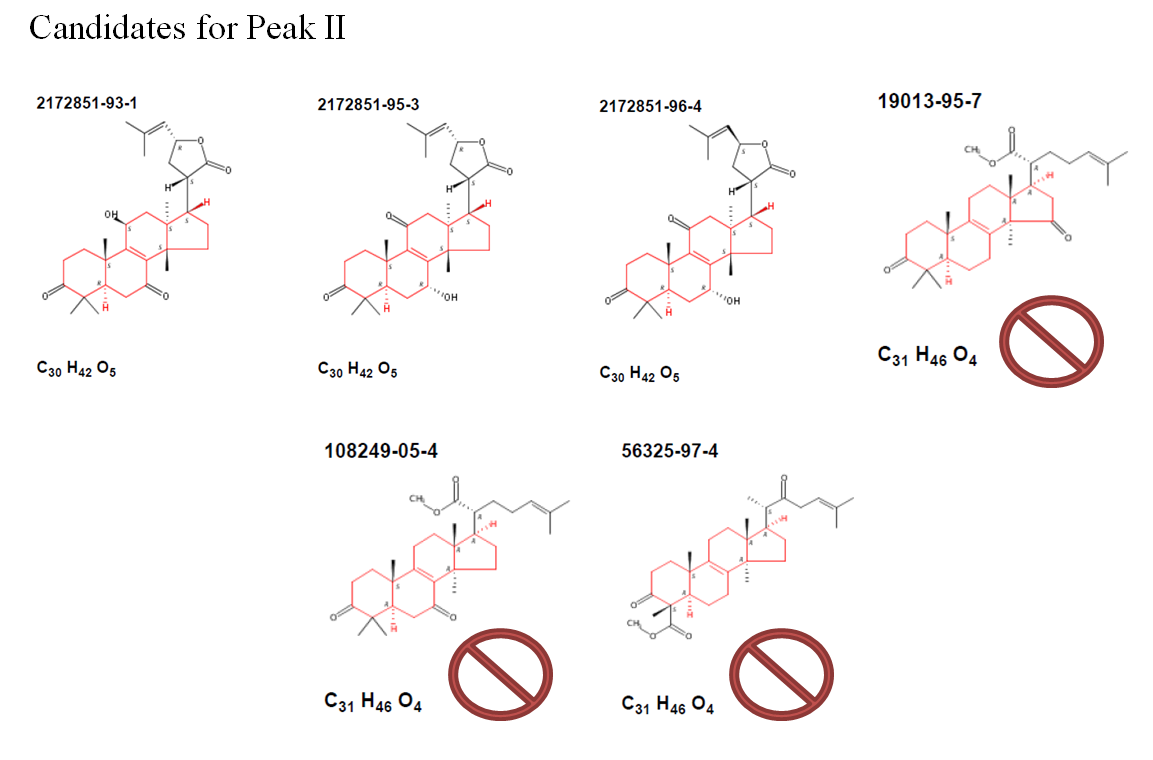


**Figure S7.** Chemical structures of all candidates for peak II. Structures with a prohibition sign were excluded after applying filters A to C.


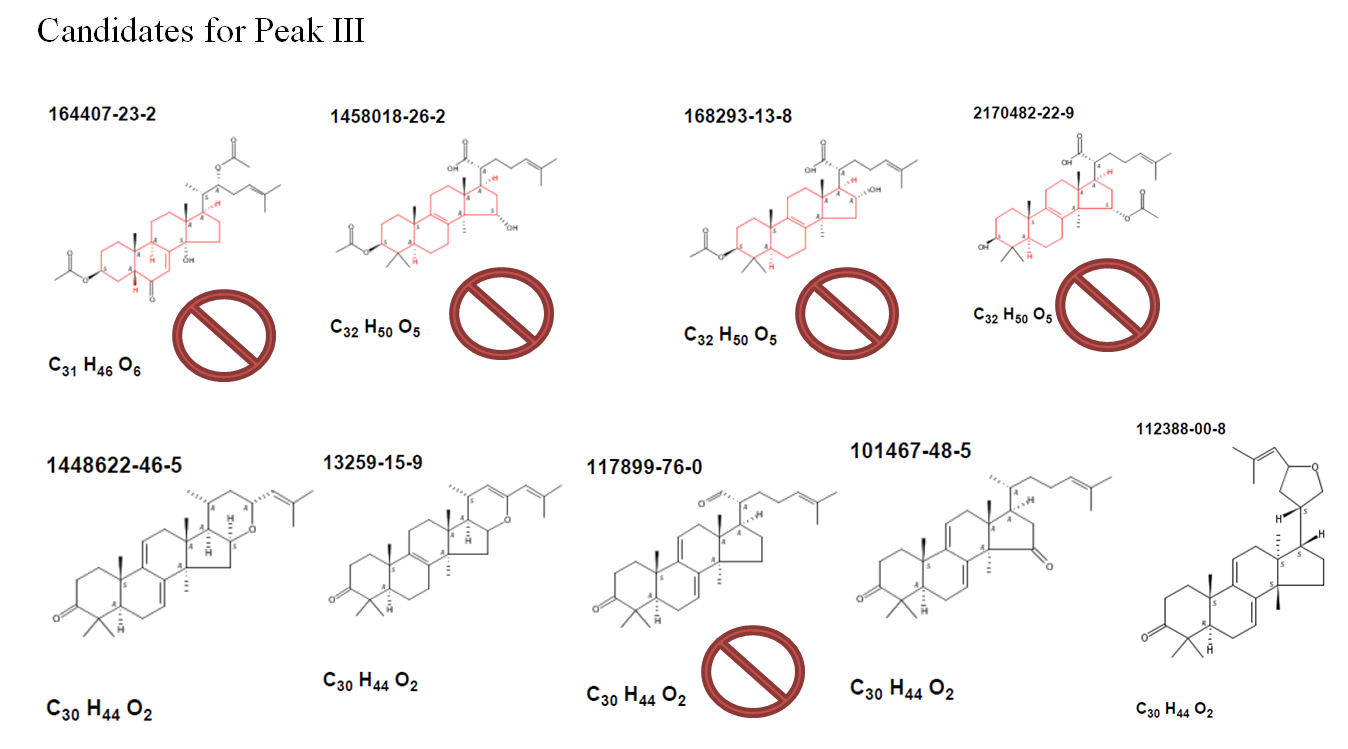


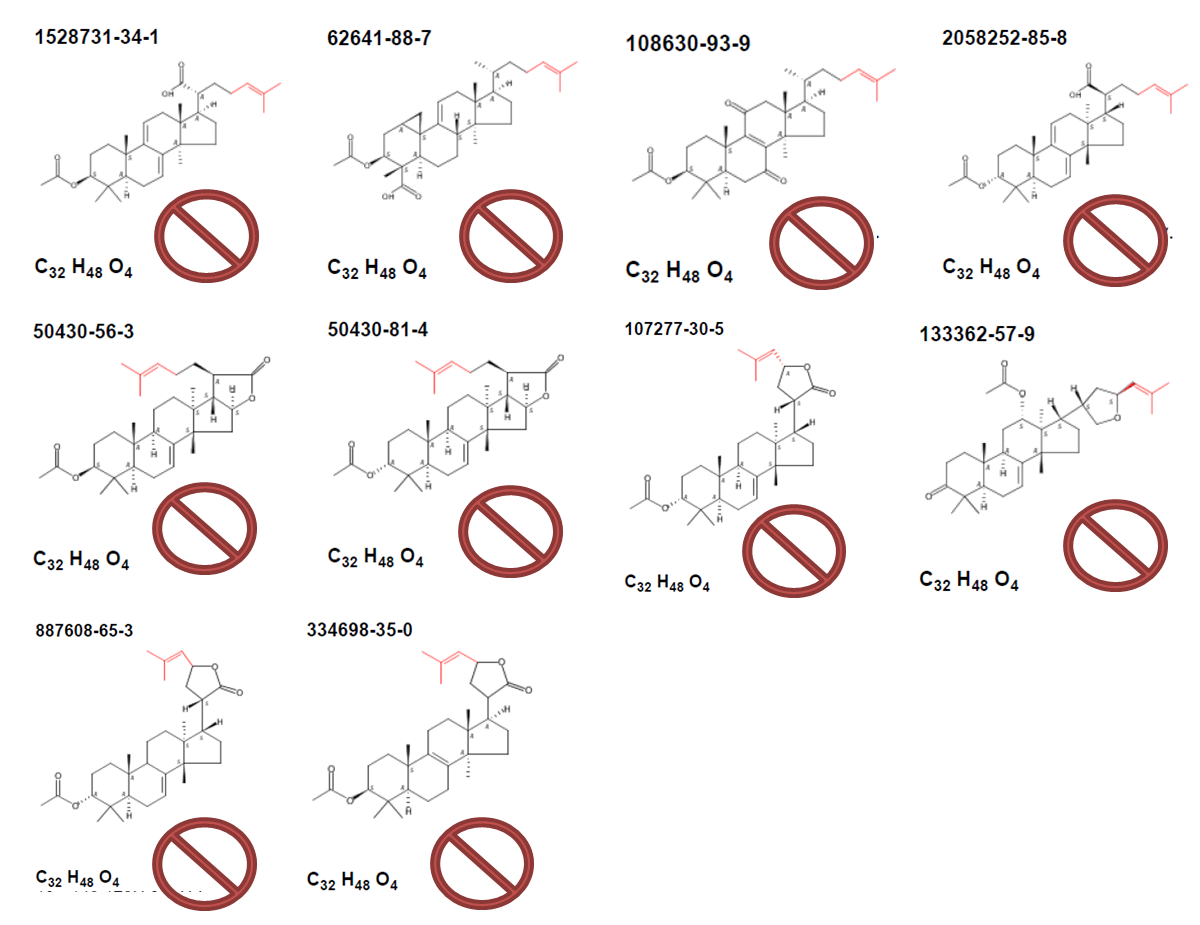


**Figure S8.** Chemical structures of all candidates for peak III. Structures with a prohibition sign were excluded after applying filters A to C.


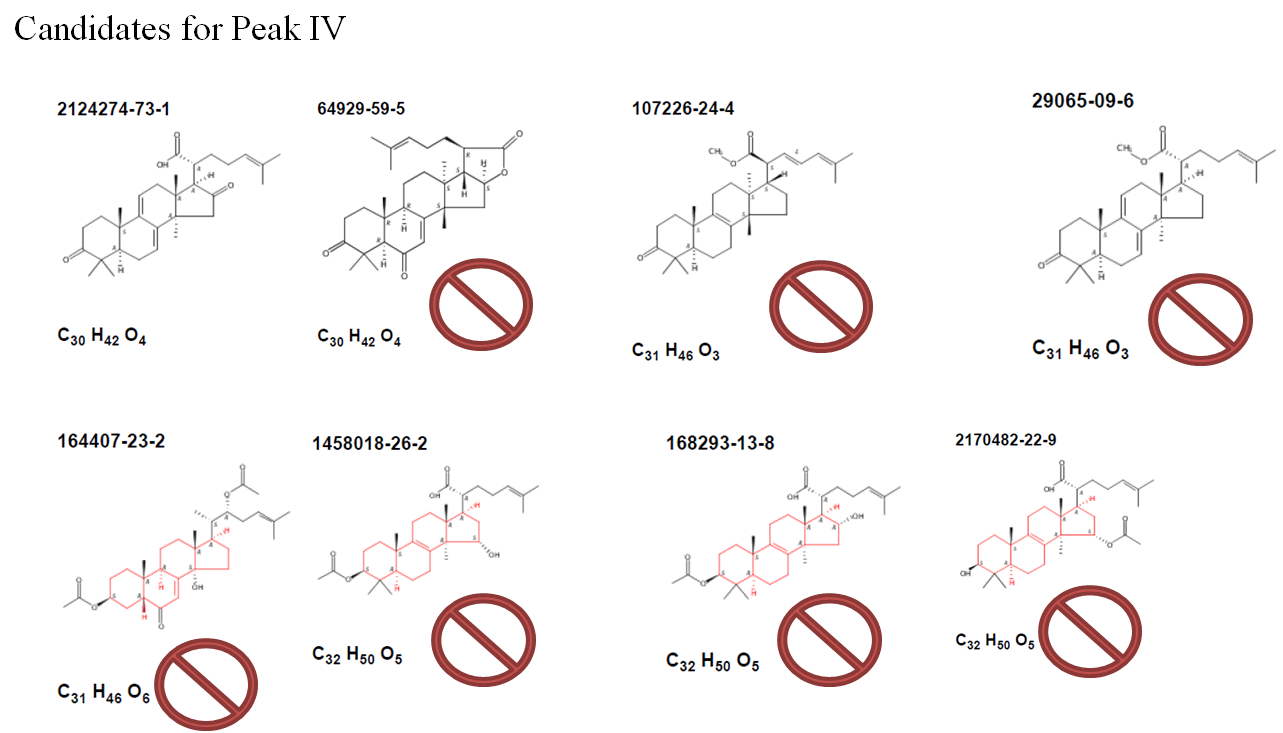


**Figure S9.** Chemical structures of all candidates for peak IV. Structures with a prohibition sign were excluded after applying filters A to C.


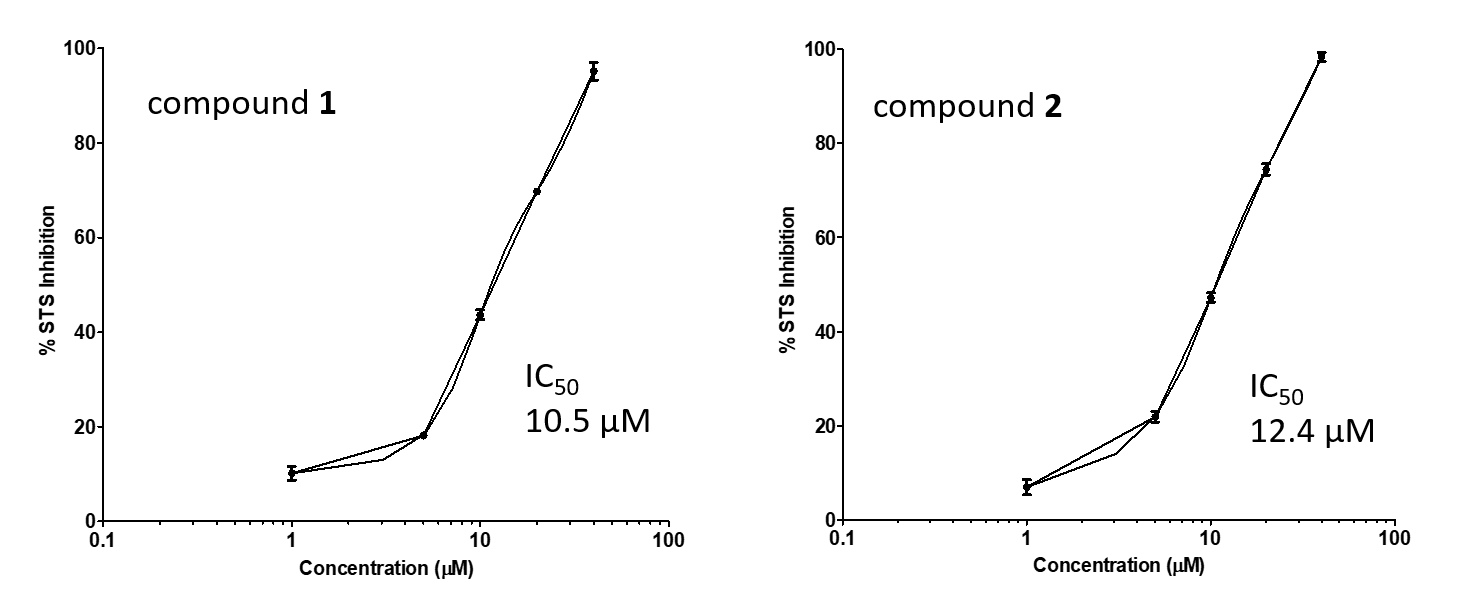


**Figure S10.** Graphs showing the concentration dependent inhibition of STS by the isolated LTTs **1**and **2** (mean ± S.D., n = 3).

Tables

**Table S1.** Overview on combined FP microfractions.

| **fraction name** | **combined tubes** | **yield [mg]** |
| --- | --- | --- |
| FP01_**01** | 3-5 | 70.11 |
| FP01_**02** | 6-8 | 22.54 |
| FP01_**03** | 9-11 | 11.18 |
| FP01_**04** | 12-24 | 14.94 |
| FP01_**05** | 25-26 | 3.59 |
| FP01_**06** | 27-29 | 5.62 |
| FP01_**07** | 30-32 | 10.4 |
| FP01_**08** | 33-35 | 4.08 |
| FP01_**09** | 36-38 | 3.50 |
| FP01_**10** | 39-43 | 6.92 |
| FP01_**11** | 44-47 | 6.93 |
| FP01_**12** | 48-50 | 9.07 |
| FP01_**13** | 51-53 | 16.00 |
| FP01_**14** | 54-56 | 22.94 |
| FP01_**15** | 57-59 | 30.31 |
| FP01_**16** | 60-62 | 11.10 |
| FP01_**17** | 63-65 | 5.22 |
| FP01_**18** | 66-68 | 9.31 |
| FP01_**19** | 69-71 | 5.45 |
| FP01_**20** | 72-74 | 4.86 |
| FP01_**21** | 75-77 | 3.83 |
| FP01_**22** | 78-80 | 11.38 |
| FP01_**23** | 81-83 | 14.50 |
| FP01_**24** | 84-86 | 65.08 |
| FP01_**25** | 87-89 | 30.63 |
| FP01_**26** | 90-92 | 22.58 |
| FP01_**27** | 93-95 | 19.36 |
| FP01_**28** | 96-98 | 5.29 |
| FP01_**29** | 99-101 | 3.58 |
| FP01_**30** | 102-104 | 3.61 |
| FP01_**31** | 105-125 | 18.15 |
| FP01_**32** | column wash | 6.88 |
| Sum | -- | 478.94 |

**Table S2.** Overview of retention times and peak areas that strongly correlate with activity.

| **peak number** | **t_R_ [min]** | **area under the curve** | | |
| --- | --- | --- | --- | --- |
|  |  | **FP01_13** | **FP01_14** | **FP01_15** |
| I | 25.2 | 174248 | 344826 | 2732908 |
| II | 25.4 | 406837 | 3714520 | 7061175 |
| III | 25.9 | 135074 | 222106 | 1225684 |
| IV | 26.2 | 279661 | 314504 | 1343099 |

**Table S3.** Overview of retention times, *m/z* values and potential molecular formula candidates that strongly correlate with activity.

| **peak number** | **t_R_ [min]** | ***m/z* [M+H]^+^** | **molecular formulas of  potential candidates** | |
| --- | --- | --- | --- | --- |
| I | 25.2 | 451.3158 | C_30_H_42_O_3_ | C_31_H_46_O_2_ |
|  |  | 425.3419 | C_29_H_44_O_2_ | C_28_H_40_O_3_ |
|  |  | 469.3615 | C_30_H_44_O_4_ | C_31_H_48_O_3_ |
| II | 25.4 | 452.3274 | C_31_H_47_O_2_ | C_30_H_43_O_3_ |
|  |  | 470.3381 | C_30_H_45_O_4_ | C_31_H_49_O_3_ |
|  |  | 483.3479 | C_31_H_46_O_4_ | C_30_H_42_O_5_ |
| III | 25.9 | 515.3738 | C_32_H_50_O_5_ | C_31_H_46_O_6_ |
|  |  | 497.3622 | C_32_H_48_O_4_ | C_31_H_44_O_5_ |
|  |  | 437.3418 | C_30_H_44_O_2_ | C_29_H_40_O_3_ |
| IV | 26.2 | 467.3529 | C_30_H_42_O_4_ | C_31_H_46_O_3_ |
|  |  | 515.3845 | C_32_H_50_O_5_ | C_31_H_46_O_6_ |
|  |  | 631.4211 | C_30_H_62_O_13_ | C_32_H_54_O_12_ |

**Table S4.** Results from the literature search in SciFinder giving the number of potential candidates for STS inhibiting LTTs after applying filters A to C.

| **peak number** | **Filter A:**  **no. of cpds with LTT backbone and hot feature of 2-methylpropenyl moiety** | **Filter B:**  **no. of cpds after excluding cold features** | **Filter C:**  **no. of cpds after excluding features that are not existing in HetCA** |
| --- | --- | --- | --- |
| I | 20 | 19 | 3 |
| II | 6 | 5 | 3 |
| III | 19 | 17 | 4 |
| IV | 8 | 6 | 1 |
| **SUM** | 53 | 47 | **11** |
